# Phase-Selective Synthesis of Copper Phosphosulfide Nanocrystals

**DOI:** 10.1021/acs.inorgchem.6c04130

**Published:** 2026-10-07

**Authors:** Haley L. Young, Danushki N. Suriyawansa, Raymond E. Schaak

**Affiliations:** Department of Chemistry, Department of Chemical Engineering, and Materials Research Institute, 8082 Pennsylvania State University, University Park, Pennsylvania 16802, United States

## Abstract

Copper phosphosulfides, including Cu_7_PS_6_ and Cu_3_PS_4_, are important materials for their ionic, electronic, and thermal conductivity properties. However, nanocrystals of these copper phosphosulfides are surprisingly rare. Here, we show that both Cu_7_PS_6_ and Cu_3_PS_4_ nanocrystals can be synthesized by reacting CuCl, P_2_S_5_, 1-dodecanethiol, and oleylamine at 250 °C for 1 h, with all reagents playing key roles in the synthesis. Phase selectivity is achieved by controlling reagent concentration, which is related to the reaction chemistry that is implicated in the formation of the nanocrystal products. Cu_7_PS_6_ forms tetrahedron-shaped nanocrystals while Cu_3_PS_4_ forms tetrapods, which can be rationalized by crystal structure relationships. The successful formation of Cu_7_PS_6_ and Cu_3_PS_4_ represents an important addition to the library of synthetically accessible nanocrystals with well-defined morphologies.

## Graphical abstract

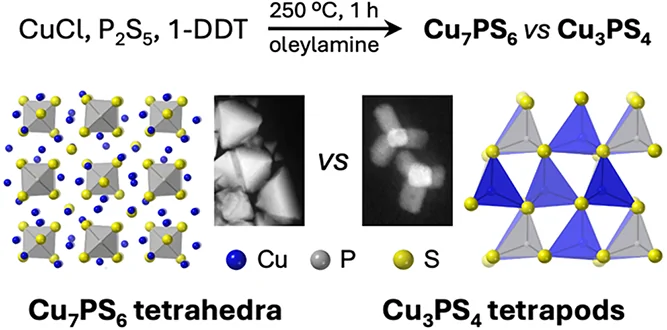

## Introduction

Ternary metal phosphosulfides, and more specifically metal thiophosphates, have electronic, optoelectronic, and catalytic properties that make them useful as nonlinear optical materials, photovoltaic absorbers, photocatalysts, electrocatalysts, and components of batteries and solar cells.^1–6^ Copper phosphosulfides, including argyrodite Cu_7_PS_6_ and enargite Cu_3_PS_4_, are especially interesting. Cu_7_PS_6_ contains isolated PS_4_ tetrahedra, along with additional isolated S^2–^ anions, surrounded by a highly disordered network of Cu^+^ cations that give rise to superionic conductivity, mixed ionic-electronic conductivity, and exceptionally low thermal conductivity.^7–9^ Cu_3_PS_4_, which is semiconducting and used for applications in batteries and solar cells, consists of an ordered three-dimensional network of corner-sharing CuS_4_ and PS_4_ tetrahedra in a wurtzite-like structure, and therefore has lower ionic conductivity than Cu_7_PS_6_.^10–14^

Bulk Cu_7_PS_6_ and Cu_3_PS_4_ are routinely synthesized using conventional high-temperature solid-state and crystal growth methods,^15,16^ as well as using alternative approaches such as mechanical milling^12^ and chemical vapor transport.^17^ However, nanocrystals of these copper phosphosulfides are surprisingly rare. Cu_3_PS_4_ nanocrystals have been synthesized by reacting Cu_3_P nanoplates with thiourea at 230 °C in oleylamine,^18^ as well as by directly combining CuCl_2_ and P_2_S_5_ powders in 1-dodecanethiol (1-DDT) at room temperature and heating to 250 °C.^19^ Nanocrystals of Cu_7_PS_6_ have not, to our knowledge, been reported.

Here, we report the synthesis of colloidal Cu_7_PS_6_ and Cu_3_PS_4_ nanocrystals. These nanocrystals form upon reacting CuCl, P_2_S_5_, 1-DDT, and oleylamine at 250 °C for 1 h, with the P_2_S_5_ reagent providing P–S linkages that favor the formation of phosphosulfides rather than competing phosphides and/or sulfides. Control over the reagent concentrations leads to phase selectivity, which emerges as a result of the complex reaction chemistry. Cu_7_PS_6_ forms tetrahedron-shaped nanocrystals while Cu_3_PS_4_ forms tetrapods; these well-defined morphologies can be rationalized based on the crystal structures.

## Experimental Section

### Chemicals

1-dodecanethiol (1-DDT, Sigma-Aldrich, ≥98%), phosphorus pentasulfide (P_2_S_5_ [also called P_4_S_10_], Sigma-Aldrich, 99%), isopropyl alcohol (IPA, Millipore), toluene (Millipore), oleylamine (Sigma-Aldrich, 70%), copper­(I) chloride (CuCl, Sigma-Aldrich, 97%). All chemicals were used as received without further purification.

### Synthesis of Cu_3_PS_4_ and Cu_7_PS_6_ Nanocrystals

Tetrapod-shaped Cu_3_PS_4_ nanocrystals were synthesized by adding 0.378 mmol CuCl and 0.189 mmol P_2_S_5_ to a 50 mL, 3-neck round-bottom flask. As the stir bar was stirring, 9.5 mL 1-DDT and 0.5 mL oleylamine were added to the flask. The reaction was kept under vacuum until the solution reached 100 °C, when it was cycled to Ar. The temperature was increased further to 250 °C and held for 1 h. The flask was then cooled to room temperature. The yellow-colored particles were precipitated using IPA, centrifuged, and resuspended in toluene three times. Tetrahedron-shaped Cu_7_PS_6_ nanocrystals were synthesized using an identical procedure to that described above for Cu_3_PS_4_, except for the amounts of CuCl (0.250 mmol) and P_2_S_5_ (0.125 mmol), which were different. The orange-colored particles were precipitated using IPA, centrifuged, and resuspended in toluene three times. For all reactions, the vacuum pressure was at or below 5 mbar and a heating rate of 15 °C/min was used. Each of these reactions was run at least five times to demonstrate reproducibility.

### Characterization

Powder X-ray diffraction (XRD) data were collected on an Empyrean diffractometer using Cu Kα radiation. XRD scans were measured from 10 to 70° 2θ with a step size of 0.02° and a scan rate of 7°/min. Zero-background Si sample holders were used for dropcasting samples. High-resolution transmission electron microscopy (HRTEM) images, high-angle annular dark field (HAADF) images, and scanning TEM coupled with energy-dispersive spectroscopy (STEM-EDS), used to acquire elemental maps, were obtained using a FEI Talos F200X S/TEM equipped with a Ceta camera and a SuperX EDS detector at an accelerating voltage of 200 kV. Samples were prepared for TEM analysis by dropcasting a dilute sample (∼0.5 mg/mL) of particles dispersed in hexanes on a Formvar-coated, 400-mesh, nickel or gold grid. Ultraviolet–visible (UV–vis) spectra were collected in a quartz cuvette using an Agilent Technologies Cary 60 UV–vis spectrophotometer. Samples were prepared in toluene and sonicated directly before measurement.

## Results and Discussion

### Phase-Selective Synthesis of Cu_3_PS_4_ and Cu_7_PS_6_

Figure 1A,B show the crystal structures of Cu_3_PS_4_ ^10^ and Cu_7_PS_6_.^7^ As discussed in the Introduction, both structures contain PS_4_ tetrahedra, but linked together in Cu_3_PS_4_ and isolated in Cu_7_PS_6_. Our initial attempts to synthesize Cu_3_PS_4_ and Cu_7_PS_6_ nanocrystals focused on using various heat-up and hot-injection methods with several sources of copper (CuCl_2_, CuCl, Cu­(acac)_2_), sulfur (1-DDT, sulfur powder), and phosphorus (trioctylphosphine, hexaethyltriaminophosphine). However, the formation of copper phosphosulfide nanocrystals competes with the formation of various stable and synthetically accessible copper sulfides and copper phosphides. Unsurprisingly in retrospect, all these attempts produced Cu_2‑x_S nanocrystals (predominantly djurleite Cu_1.94_S) instead of the desired copper phosphosulfides, in part because the temperature at which Cu_2‑x_S nanocrystals form is well below that of both the copper phosphosulfides and Cu_3‑x_P. Considering that Cu_7_PS_6_ and Cu_3_PS_4_ contain [PS_4_]^3–^ polyanion units, we pivoted to a simple heat-up synthesis using P_2_S_5_ as a reagent, inspired by a prior report^19^ of Cu_3_PS_4_ nanocrystal synthesis and motivated by the presence of P–S linkages in the precursor. Our reaction involved the addition of CuCl and P_2_S_5_, with a 1:1 Cu:P ratio, to a round-bottom flask, followed by the addition of 9.5 mL of 1-DDT and 0.5 mL of oleylamine. The resulting solution was heated under Ar to 250 °C and then held at that temperature for 1 h before cooling to room temperature and isolating the nanocrystal product. It is important to note that simultaneously mixing CuCl and P_2_S_5_ in 1-DDT was crucial for avoiding the formation of copper sulfide impurities, as discussed in detail in a later section. A hot-injection synthesis using P_2_S_5_ was not attempted because P_2_S_5_ is insoluble in 1-DDT below 160 °C.^19^

**Figure 1:**
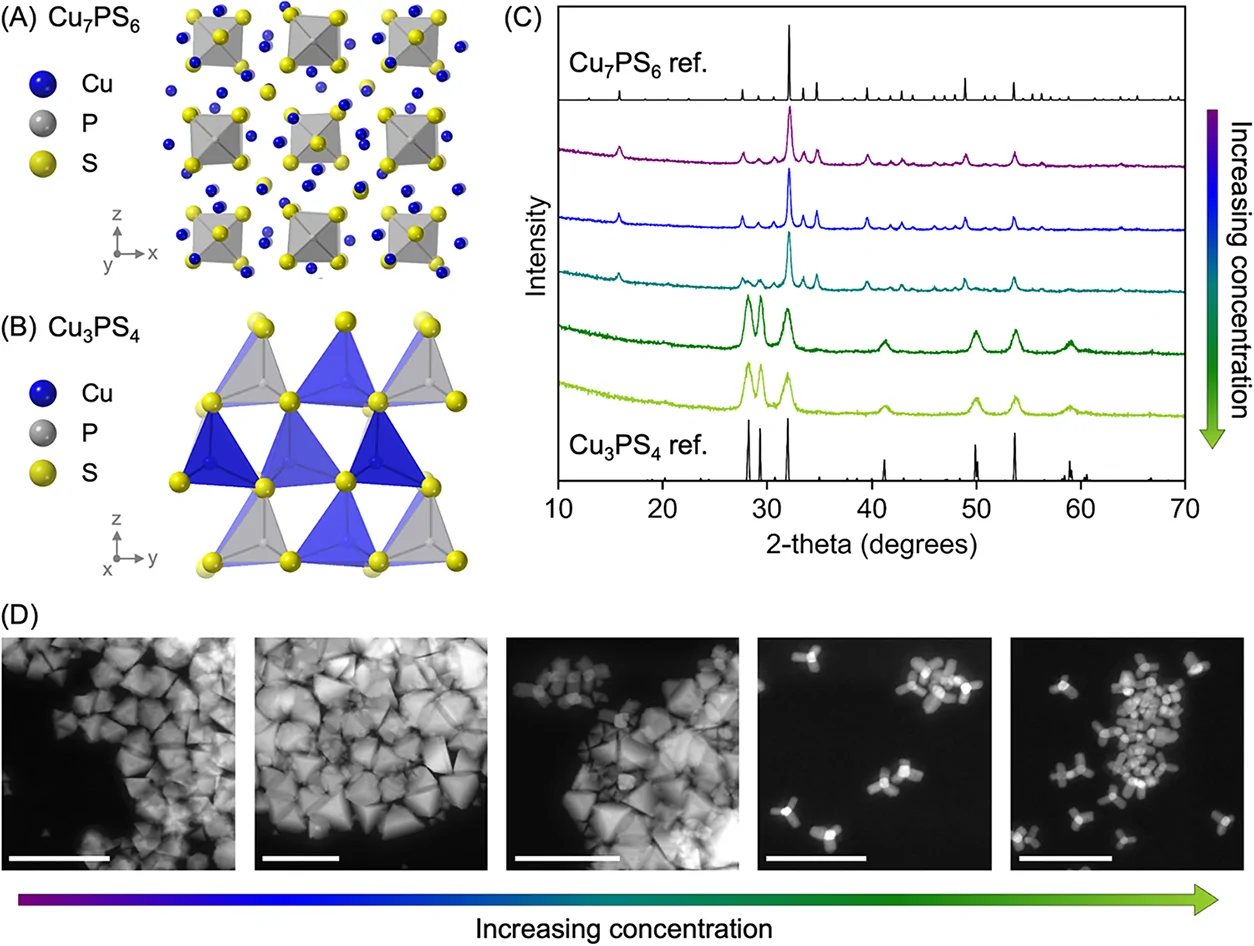
Crystal structures of (A) Cu_7_PS_6_ and (B) Cu_3_PS_4_, highlighting the [PS_4_]^3–^ tetrahedral units as polyhedra. The reference XRD patterns for Cu_7_PS_6_ and Cu_3_PS_4_ are from ref 15 and ICSD 98-041-2240, respectively. (C) XRD patterns and (D) STEM images of the copper phosphosulfide products at different reaction concentrations, as described in the main manuscript text. Scale bars are 100 nm.

We initially began by using 0.125 mmol of P_2_S_5_ and 0.250 mmol of CuCl to maintain a 1:1 Cu:P ratio, which was used in the prior reported synthesis of Cu_3_PS_4_ nanocrystals. Figure 1C includes the powder XRD pattern for the product isolated from this reaction, which matches well with the reference pattern for Cu_7_PS_6_. Despite loading a 1:1 Cu:P ratio, the product that formed is highly enriched in copper, suggesting either that not all the P_2_S_5_ is available to react or that Cu_7_PS_6_ simply forms first. To explore these possibilities further, we carried out a series of experiments that maintained the 1:1 Cu:P molar ratio in 9.5 mL of 1-DDT and 0.5 mL of oleylamine but varied the concentrations of CuCl and P_2_S_5_. A lower-concentration reaction with 0.0625 mmol of P_2_S_5_ and 0.125 mmol of CuCl also produced Cu_7_PS_6_ (Figure 1C). A higher-concentration reaction involving 0.157 mmol of P_2_S_5_ and 0.314 mmol of CuCl still formed Cu_7_PS_6_ as the majority product (Figure 1C), but additional low-intensity peaks at 28° and 29.5° 2θ match with the two most intense reflections of Cu_3_PS_4_. Increasing the concentrations further to 0.378 mmol of CuCl (with 0.189 mmol of P_2_S_5_) and 0.500 mmol of CuCl (with 0.250 mmol of P_2_S_5_) produces exclusively Cu_3_PS_4_, based on XRD analysis (Figure 1C). STEM-EDS data, along with the corresponding EDS spectra, confirm the colocalization of Cu, P, and S in the expected ratios within the Cu_7_PS_6_ and Cu_3_PS_4_ nanocrystals (Figure S1). Additionally, Figure S2 shows UV–visible absorption spectra and corresponding Tauc plots for the Cu_7_PS_6_ and Cu_3_PS_4_ nanocrystals, which exhibit optical band gaps of 2.4 and 2.6 eV, respectively. These values are consistent with the orange color of Cu_7_PS_6_ and yellow color of Cu_3_PS_4_ observed for the corresponding colloidal nanoparticle suspensions, as well as literature values.^11,20,21^

Although not necessarily anticipated in advance, the absolute concentrations of the reactants, while maintaining a constant Cu:P ratio of 1:1, played a key role in the formation of Cu_3_PS_4_ versus that of Cu_7_PS_6_. We note that the higher-concentration reactions formed Cu_3_PS_4_, which is more phosphorus-rich than Cu_7_PS_6_; therefore, we rationalize this concentration-dependent phase selectivity based on precursor speciation and reaction kinetics. Under these reaction conditions, we would expect CuCl to form oleylamine-solvated Cu­(I) complexes and Cu­(I)-dodecanethiolate species, while P_2_S_5_ would react with oleylamine and 1-DDT to form soluble thiophosphate species. At higher concentrations, the reactive phosphorus-containing species could promote the formation of Cu–S–P complexes prior to Cu­(I)-thiolate decomposition, favoring the more phosphorus-rich Cu_3_PS_4_ phase. At lower precursor concentrations, the higher ligand-to-precursor ratio would be expected to stabilize the Cu­(I)-dodecanethiolate complex, promoting the formation of intermediates rich in Cu–S-based extensive phosphorus incorporation, thereby limiting phase formation to the more copper-rich Cu_7_PS_6_ phase.

HAADF-STEM images for all five samples from Figure 1C are shown in Figure 1D. The two samples that had XRD patterns showing only Cu_7_PS_6_ products appear in Figure 1D as polydisperse particles having diameters ranging from 5 nm up to beyond 50 nm and morphologies that can be described as tetrahedra, prisms, and triangular plates. All these morphologies are consistent with the crystal structure of Cu_7_PS_6_, as shown for comparison in Figure 1A. In contrast, the Cu_3_PS_4_ nanocrystals form tetrapods having a more uniform size with diameters near 25 nm. This morphology is also consistent with the crystal structure of Cu_3_PS_4_ (Figure 1B). For the middle sample, which was mostly Cu_7_PS_6_ by XRD with a minor contribution from Cu_3_PS_4_, tetrapods of Cu_3_PS_4_ are observed to coexist with tetrahedra of Cu_7_PS_6_.

The formation of Cu_3_PS_4_ tetrapods here is therefore notable because the literature report that inspired our use of the P_2_S_5_ reagent reported Cu_3_PS_4_ nanocrystals having irregular morphologies.^19^ Our ability to select between Cu_7_PS_6_ and Cu_3_PS_4_ was due to the molar ratios of the CuCl and P_2_S_5_ reagents, but to understand these differences in morphology for Cu_3_PS_4_, we need to consider the overall reagent concentration. The report that produced irregularly shaped Cu_3_PS_4_ nanocrystals used significantly higher concentrations of reactants (2 mmol of Cu reagent in 4 mL of solvent),^19^ whereas our reaction that produced Cu_3_PS_4_ tetrapods used more dilute reagents (0.250 mmol of Cu reagent in 10 mL of solvent). To understand how reagent concentration may impact Cu_3_PS_4_ nanocrystal morphology, we emulated a higher-concentration reaction by decreasing the amount of solvent to 4.0 mL while keeping a 1:1 ratio of Cu:P. The resulting Cu_3_PS_4_ nanocrystals were indeed irregularly shaped, as shown in the HAADF-STEM image in Figure 2A. We suspect that the higher concentrations facilitate more rapid, nonspecific, and self-limiting growth of the nanocrystals, forming small nanocrystals with irregular shapes, while lower concentrations slow growth so that the [111] facets grow preferentially, leading to larger tetrapods. Figure 2B shows that the XRD pattern of the sample containing irregularly shaped particles has significantly broader peaks than the Cu_3_PS_4_ tetrapods, which is consistent with the smaller crystallite size.

**Figure 2:**
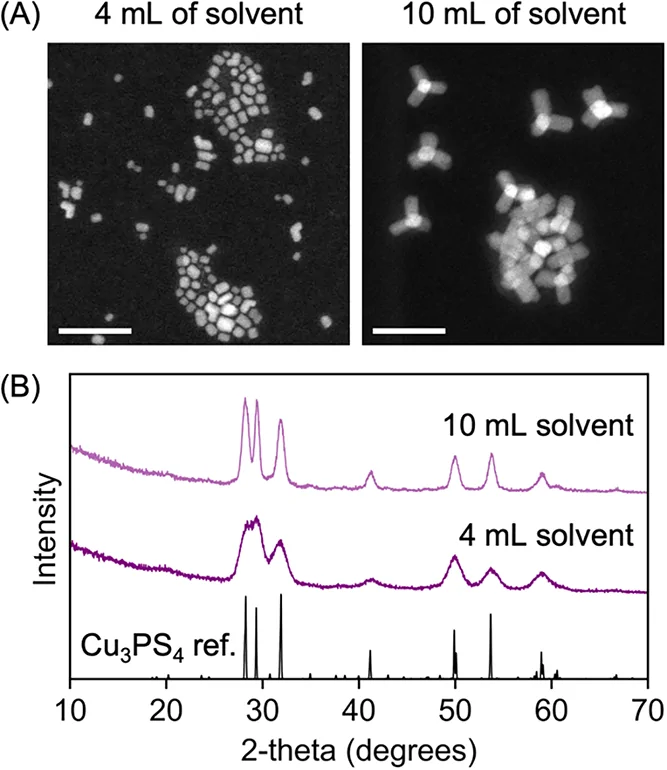
Comparison of the (A) STEM images and (B) XRD patterns of Cu_3_PS_4_ when 0.378 mmol CuCl and 0.189 mmol P_2_S_5_ are loaded into 4 mL of solvent versus 10 mL of solvent. Differences in nanocrystal shape and size are observed. Scale bars are 50 nm.

### Insights into Nanocrystal Formation

To better understand how the Cu_7_PS_6_ and Cu_3_PS_4_ nanocrystals grow, we carried out aliquot studies from the reactions using 0.250 mmol CuCl and 0.378 mmol CuCl, respectively. After the reaction reached 250 °C, aliquots were taken at 15 min, 30 min, 45 min, 1, 1.5, and 2 h. The XRD patterns and HAADF-STEM images for each of the aliquots are shown in Figure 3. Looking first at the time-dependent XRD data for Cu_7_PS_6_ in Figure 3A, Cu_7_PS_6_ is present already at 15 min. In this 15 min sample, the XRD pattern has a lower signal-to-noise ratio and broader peaks, which indicates that the nanocrystals have, on average, smaller crystalline domain sizes, compared to the products extracted from later aliquots. The corresponding HAADF-STEM image of the 15 min aliquot sample shows primarily 5–10 nm particles with a tetrahedral shape, along with a smaller subpopulation of larger ∼ 50 nm particles. As the reaction progresses from 30 min to 2 h, XRD analysis of the aliquots shows that the Cu_7_PS_6_ phase persists. The corresponding HAADF-STEM images in Figure 3B show that the larger particles increase in size while the smaller tetrahedra decrease. Note that tetrapod-shaped particles that likely correspond to Cu_3_PS_4_ are occasionally observed in the HAADF-STEM images for the 15-, 30-, and 45 min aliquots, but not enough to show up by XRD. In the 1-, 1.5-, and 2-h aliquots, there is no evidence that the tetrapods are still present. The colors observed for each of the aliquots, also shown in Figure 3A, are qualitatively consistent with what we would expect based on the XRD and HAADF-STEM data. At early time points, the color is consistent with the orange Cu_7_PS_6_ nanocrystals mixed with the yellow color associated with Cu_3_PS_4_, transforming to the orange color associated with Cu_7_PS_6_ at longer time points.

**Figure 3:**
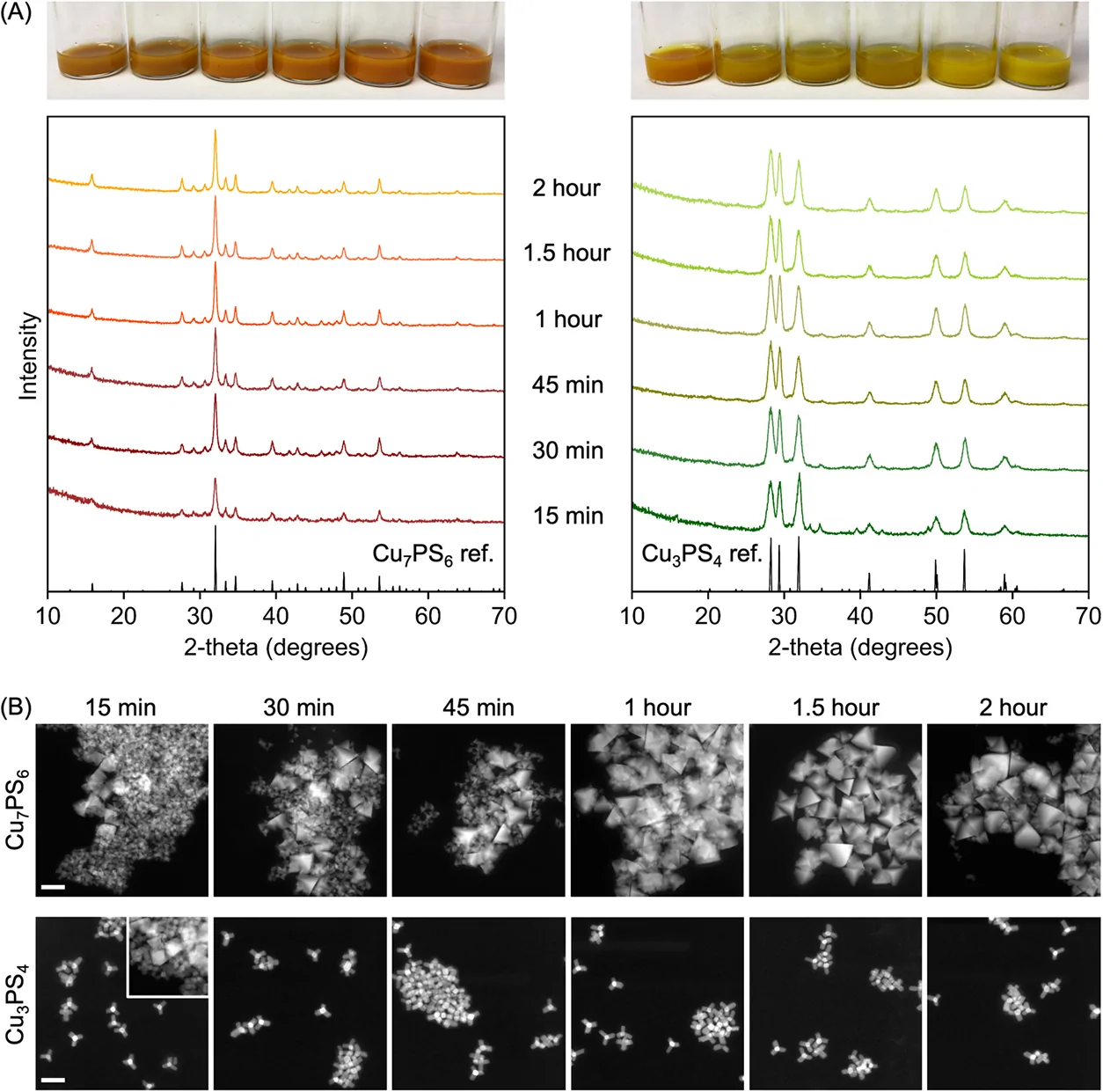
(A) XRD patterns from the aliquot study of Cu_7_PS_6_ and Cu_3_PS_4_, showing the products extracted at time points ranging from 15 min to 2 h. Photographs of vials containing each aliquot are pictured above the XRD patterns; each series of vials is arranged from the 15 min time point on the left to the 2-h time point on the right. (B) STEM images for Cu_7_PS_6_ and Cu_3_PS_4_ samples isolated from the aliquot study, corresponding to the series of XRD patterns in panel (A). The image in the inset for the 15 min Cu_3_PS_4_ sample shows evidence for a Cu_7_PS_6_ subpopulation, based on morphology comparisons, in the 15 min aliquot. Scale bars, which are the same for all STEM images, are 50 nm.

Figure 3A also shows XRD data for the Cu_3_PS_4_ aliquots. The XRD pattern of the 15 min aliquot matches the reference pattern for Cu_3_PS_4_, along with several impurity peaks that match well with the most intense peaks for Cu_7_PS_6_. This result is not surprising, given the observation in Figure 1C that Cu_7_PS_6_ forms readily. Consistent with the observation of a Cu_7_PS_6_ subpopulation at 15 min, the HAADF-STEM image from the 15 min aliquot (Figure 3B) shows the ∼25 nm tetrapods that correspond to Cu_3_PS_4_ and an additional subpopulation of prismatic particles that are consistent with the Cu_7_PS_6_ nanocrystals. As the reaction progresses to 30 min and beyond, the XRD peaks corresponding to the Cu_7_PS_6_ phase disappear. The HAADF-STEM images similarly reflect the disappearance of the large prismatic Cu_3_PS_4_ particles, leaving exclusively monodisperse ∼25 nm tetrapods of Cu_3_PS_4_. In both aliquot studies, the expected copper phosphosulfide phases were present as majority phases at the 15 min time points, along with minority subpopulations of the other copper phosphosulfide. By the end of the reactions, the minority subpopulations were no longer observed. Collectively, these observations suggest that Cu_3_PS_4_ and Cu_7_PS_6_ form together early in the reaction, with the Cu:P ratio ultimately dictating how the reaction evolves and which phases ultimately form in highest yield. These observations are also consistent with the colors of the aliquots, also shown in Figure 3A. At early time points, the yellow-orange color matches well with what we would expect for a mixture of the orange Cu_7_PS_6_ and yellow Cu_3_PS_4_ nanocrystals, transforming to predominantly yellow Cu_3_PS_4_ nanocrystals at longer time points.

The STEM images in Figure 3B showed that the Cu_3_PS_4_ tetrapod products remained unchanged across all aliquots from 15 min to 2 h. To probe how the tetrapods formed, we carried out a second aliquot study at earlier time points of 1, 3, and 5 min into the reaction. As shown in Figure 4, at 1 min into the reaction, small particles are observed, most having diameters of 10 nm or less. Some of these particles appear to consist of aggregates of even smaller particles, while others show evidence of armlike features that are reminiscent of tetrapods. At 3 min, most of the particles adopt tetrahedral shapes that are reminiscent of the Cu_7_PS_6_ nanocrystals; additional tetrapod-shaped nanocrystals, which we attribute to Cu_3_PS_4_, are also present. By 5 min, the majority of the particles are tetrapods. This type of *ex situ* study applied to a system that forms so soon after the initiation of a high-temperature colloidal reaction is unable to deconvolute various growth stages to unambiguously assign a detailed mechanism; a dedicated *in situ* study would be needed. Nonetheless, the data suggest that tetrahedrally shaped Cu_7_PS_6_ nanocrystals may form first and seed the growth of Cu_3_PS_4_ tetrapods. This mechanistic hypothesis is supported by the crystal structure considerations. Cu_3_PS_4_ forms a wurtzite-like structure, and tetrapod morphologies are common among wurtzite- and wurtzite-like nanocrystals.^22–24^ Tetrapod morphologies in such nanocrystals often initiate through the formation of octahedral or tetrahedral seed nuclei,^24–26^ such as those observed here at the earlier time points.

**Figure 4:**
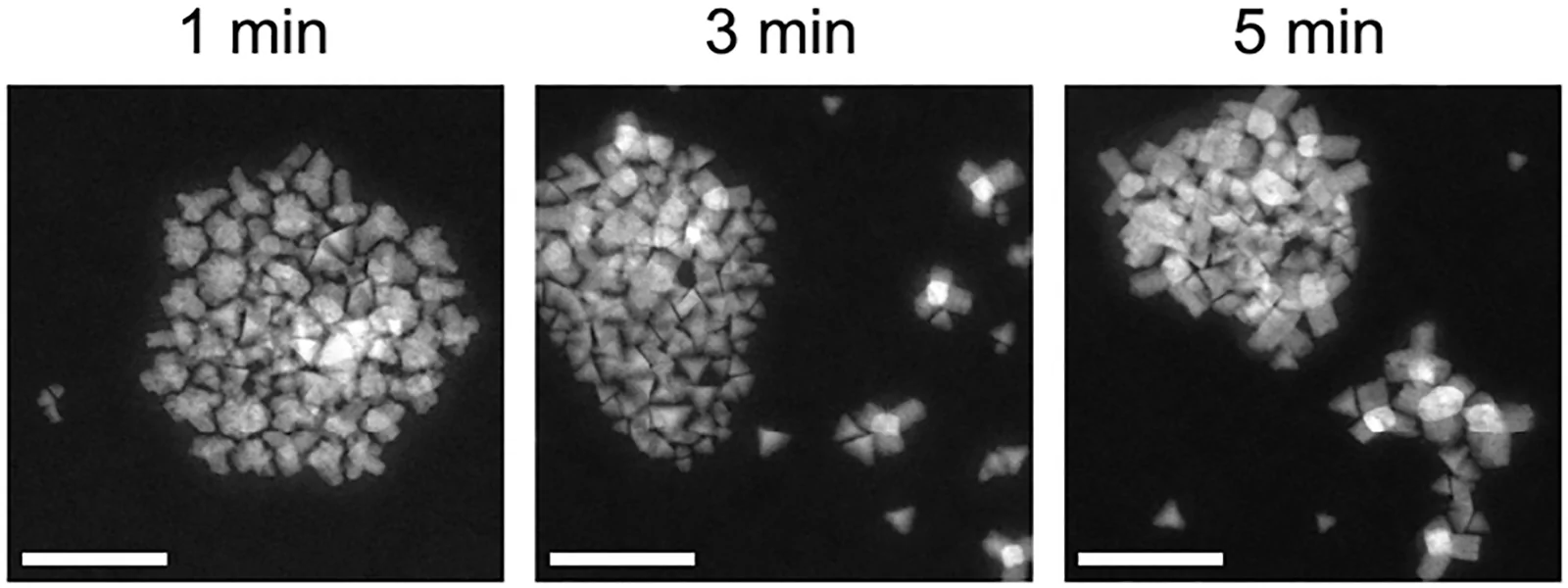
STEM images of early time points of the reaction that forms Cu_3_PS_4_ nanocrystals. Scale bars are 50 nm.

On a TEM grid, the Cu_3_PS_4_ tetrapods preferentially orient with one arm perpendicular to the grid. High-resolution transmission electron microscopy (HRTEM) was used to resolve the growth direction of the tetrapod arms (Figure 5A). The orientation of the center arm clearly resolves the (010) and (230) planes, identified by the *d*-spacing values of 6.35 Å and 1.84 Å, respectively, that were determined by measuring and averaging more than 5 individual lattice planes per particle. This value closely matches the actual values of 6.33 Å and 1.82 Å from the Cu_3_PS_4_ crystal structure. A depiction of the Cu_3_PS_4_ sulfur sublattice is shown to visualize the orientation of the measured planes; a row of sulfur atoms is highlighted in green to help track the orientation of the sublattice upon rotation to match the observation direction. HRTEM images of different Cu_3_PS_4_ tetrapods (Figure S3) show the crystallinity of the arms that are oriented more horizontally on the TEM grid. Figure 5B shows an HRTEM image of a representative Cu_7_PS_6_ nanocrystal. These nanocrystals aggregate more than the tetrapods, making it challenging to isolate a single particle for imaging. The image shown here focuses on the edge of a particle where the (2̅02) and (022̅) planes are identified by their measured *d*-spacings of 3.50 Å and 3.45 Å, which agree well with the actual value of 3.42 Å for both. The particle is oriented so that the face of the (111) plane is resolved, as represented by the green-highlighted S atoms.

**Figure 5:**
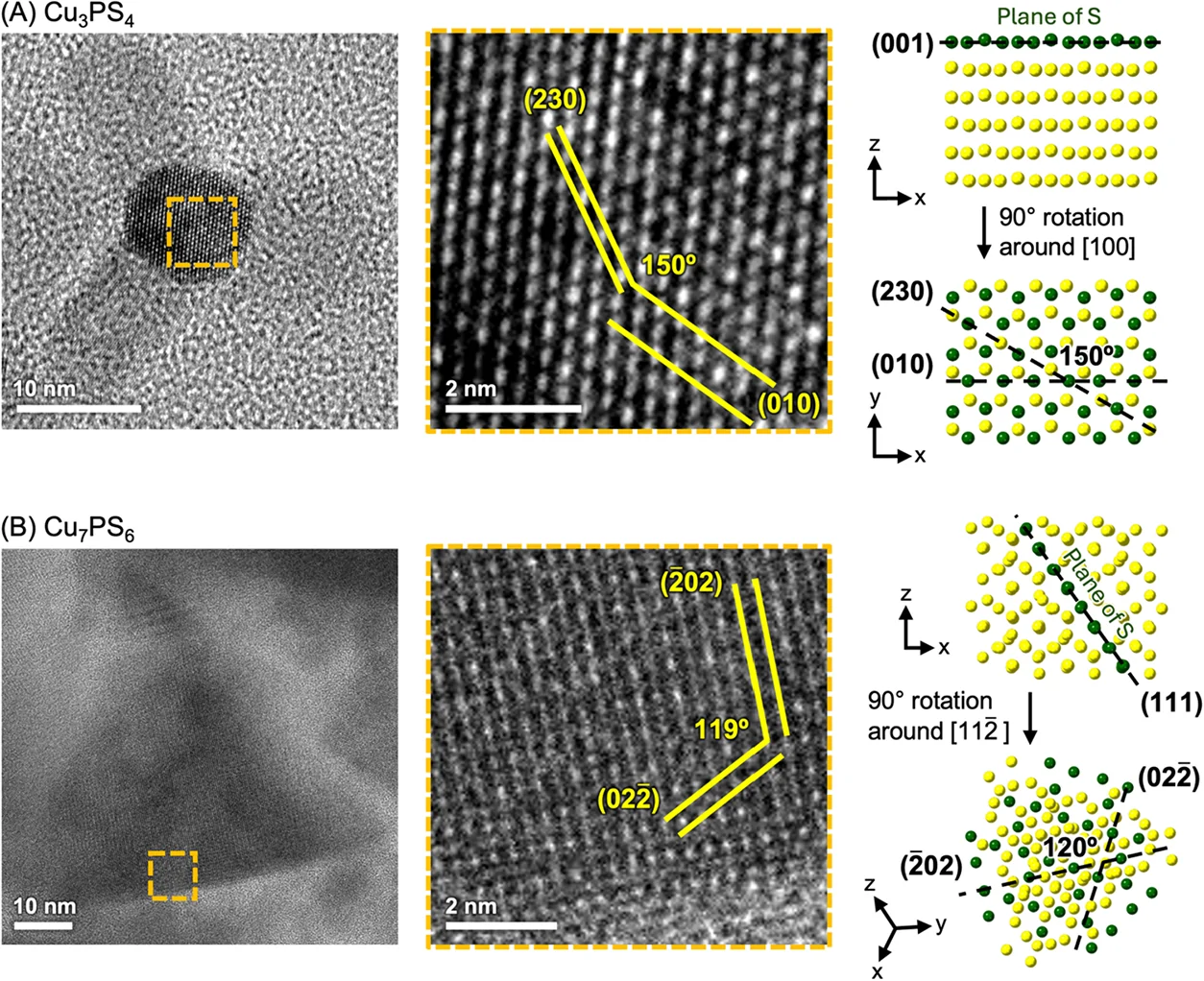
HRTEM images of (A) a Cu_3_PS_4_ tetrapod and (B) a prism-shaped Cu_7_PS_6_ nanocrystal, along with enlarged regions as indicated by the dashed orange boxes. The crystal structures of the sulfur sublattice (yellow) are also shown, along with the planes identified in the HRTEM image, as labeled. The green atoms highlight a single plane of S atoms, in different orientations. The *d*-spacings and angles measured from the HRTEM image match well with the actual values expected based on the crystal structure.

### Insights into Reaction Chemistry

To learn more about the reaction chemistry that leads to the formation of Cu_3_PS_4_ and Cu_7_PS_6_ nanocrystals using CuCl, P_2_S_5_, 1-DDT, and oleylamine, we returned to further investigate the Cu:P ratio, beyond the experiments initially carried out as we were developing the phase-selective synthesis. We also studied the roles of 1-DDT and oleylamine in greater detail to provide a greater understanding of the key components of this nanochemical reaction.

We first considered the effect of the Cu:P molar ratio, starting from the reaction conditions where the Cu:P ratio was 1:1 and the P_2_S_5_ loading was either 0.125 or 0.189 mmol. As described above, the products formed by using these reaction conditions were Cu_7_PS_6_ and Cu_3_PS_4_, respectively. In these reactions, copper is the limiting reagent based upon a comparison of the reagent amounts and the copper phosphosulfide stoichiometries. To begin studying how the Cu:P ratio influences the product phase and yield, we kept the P_2_S_5_ loading constant and subsequently changed the amount of CuCl to reflect Cu:P ratios of 3:1 and 7:1. These ratios were chosen to match the stoichiometric ratio of Cu:P in the expected Cu_3_PS_4_ and Cu_7_PS_6_ products, respectively. The STEM images and XRD patterns of the resulting products are shown in Figure 6. When a Cu:P ratio of 3:1 was used with a loading of 0.125 mmol P_2_S_5_, the product was predominately prism-shaped Cu_7_PS_6_ nanocrystals with a subpopulation of hexagonal plates, which are attributed to copper sulfide impurities that are present in quantities that are too small to detect by XRD. When the same Cu:P ratio of 3:1 was used with a loading of 0.189 mmol of P_2_S_5_, the XRD pattern showed primarily Cu_7_PS_6_ along with a minor Cu_3_PS_4_ impurity.

**Figure 6:**
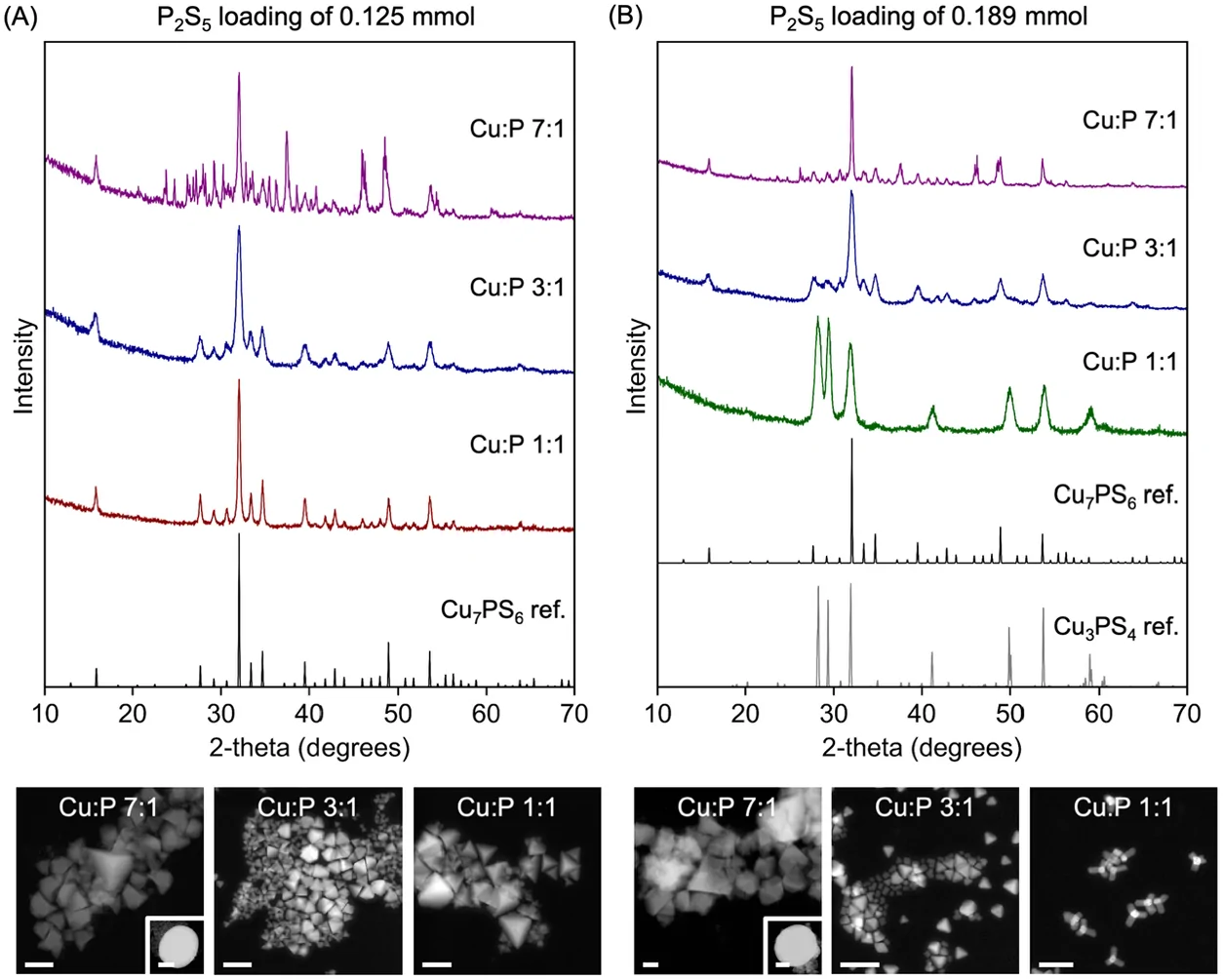
XRD patterns and STEM images of the products when a Cu:P ratio of 1:1, 3:1, and 7:1 is used with a P_2_S_5_ loading of (A) 0.125 mmol and (B) 0.189 mmol. Peaks not accounted for by the Cu–P–S phases correspond to djurleite copper sulfide (Figure S4). Scale bars for the main STEM images are 50 nm. Inset images show the large micron-sized particulates with scale bars of 200 nm.

When a Cu:P ratio of 7:1 was used under both conditions (0.125 or 0.189 mmol P_2_S_5_), dark brown and olive-colored products were formed, which contrasts with the typical orange color of Cu_7_PS_6_ and yellow color of Cu_3_PS_4_ when a Cu:P ratio of 1:1 is used. XRD data for the products showed a mixture of djurleite copper sulfide (Cu_1.94_S) and Cu_7_PS_6_ (Figure S4), which is consistent with the reaction chemistry. 1-DDT is commonly used as a sulfur source for the synthesis of binary copper sulfide nanocrystals.^27^ Given the large amount of CuCl relative to P_2_S_5_, the CuCl likely reacts with 1-DDT before reacting with the P_2_S_5_. As mentioned earlier, the formation temperature of copper sulfide (≤200 °C) is well below that of copper phosphosulfide (∼250 °C). The STEM images in Figure 6 show the irregularly shaped Cu_7_PS_6_ particles as well as larger micron-sized particles, consistent with the sharp peaks observed by XRD.

To summarize the influence of stoichiometry on product formation, a 1:1 Cu:P loading ratio is needed to access Cu_3_PS_4_ and Cu_7_PS_6_, with no impurities observed by XRD. For both systems, however, increasing the copper loading leads to the formation of Cu_7_PS_6_, which is the more copper-rich phosphosulfide phase. At even higher copper loadings, additional copper-rich copper sulfides also form. While the formation of copper sulfide from CuCl is a competing reaction, there are likely also competing reactions with P_2_S_5_. It is known that P_2_S_5_ dissolves in 1-DDT and forms various phosphorus species in solution;^19,28^ we suspect that these phosphorus species are unreactive toward the formation of copper phosphosulfides.

Next, we considered the roles of 1-DDT and oleylamine. In the direct synthesis of many metal sulfide nanocrystals, 1-DDT is commonly used as a sulfur reagent, a stabilizing ligand, or a solvent.^29,30^ In many cases, the thermal decomposition of 1-DDT is initiated by a reaction of the thiol group with the metal cations, forming a metal thiolate.^31^ As discussed above, the use of excess 1-DDT likely contributes to the competing formation of copper sulfide. We considered the possibility that copper sulfide forms early in the reaction and subsequently undergoes phosphorization from the decomposition products of P_2_S_5_ to give the copper phosphosulfide products. However, this possible formation pathway is unlikely based on visual observations of the reaction as it progressed. For all reactions, the color progression begins as a turbid, golden yellow color when 1-DDT is added to the flask containing P_2_S_5_ and CuCl, which is expected based on the color and insolubility of P_2_S_5_ under these conditions. As the flask is heated, the solution turns translucent yellow/orange due to the P_2_S_5_ dissolving. This observation is consistent with prior reports that P_2_S_5_ fully dissolves in 1-DDT around 160 °C.^19^ The reactions then transition ultimately to the expected colors of Cu_3_PS_4_ (yellow) and Cu_7_PS_6_ (orange), similar to the progression of aliquots shown in Figure 3A. If copper sulfide nanoparticles were forming during this reaction in appreciable amounts, we would have expected to observe their characteristic dark brown color, which was not the case.

During the reaction, 1-DDT is added to the reactants followed by oleylamine. Prior work on the synthesis of Cu_3_PS_4_ nanocrystals described the simultaneous mixing of P_2_S_5_ with the copper reagent as being crucial to avoiding other reaction products.^19^ P_2_S_5_ is known to react with thiols to form thiophosphate esters, which readily dissolve in any remaining excess thiol.^22^ A prior study proposed that P_2_S_5_ dissolves in 1-DDT and forms two types of phosphorus species. One of the phosphorus species was predicted to be a solvated form of P_2_S_5_ in 1-DDT that forms at lower temperatures and reacts with the copper source in solution, leading to the formation of copper phosphosulfide. Hence, simultaneous addition of P_2_S_5_ and CuCl would be expected to be crucial for forming the copper phosphosulfide nanocrystals. The other phosphorus species was predicted to be a group of byproducts from the solvation of P_2_S_5_ in 1-DDT, possibly (RS)_3_PS and (RS)_2_PSSH.^31^ At elevated temperatures, these species were more prevalent and could lead to a mixture of products if the copper source was not directly added with the P_2_S_5_.

Oleylamine is a long alkyl-chain amine that is commonly used as a solvent and stabilizing ligand in nanocrystal synthesis.^32,33^ The color of the copper phosphosulfide reaction solution changed from golden yellow to neon yellow after adding the oleylamine to 1-DDT, P_2_S_5_, and CuCl. This observation suggests that oleylamine plays an important role in the formation of the reaction precursors, possibly coordinating to relevant phosphorus or copper species. To investigate the importance of oleylamine in the reaction, we replicated the synthesis of Cu_7_PS_6_ and Cu_3_PS_4_ without oleylamine being present (Figure 7). Traditionally, a loading of 0.250 mmol of CuCl and 0.125 mmol of P_2_S_5_ with 9.5 mL of 1-DDT and 0.5 mL of oleylamine yields prism-shaped Cu_7_PS_6_ nanocrystals, while a loading of 0.378 mmol of CuCl and 0.189 mmol of P_2_S_5_ yields Cu_3_PS_4_ tetrapods. The XRD patterns obtained from these products, originally shown in Figure 1C, are shown again in Figure 7, along with the XRD patterns for the products obtained without oleylamine. When oleylamine was excluded, the product formed for both procedures was Cu_3_PS_4_; Cu_7_PS_6_ was not observed without oleylamine being present. The STEM images show that the Cu_3_PS_4_ nanocrystals formed without oleylamine adopt irregular dendritic morphologies. Oleylamine therefore appears to be important for growing discrete nanocrystals and stabilizing the growth of preferential facets that lead to their well-formed morphologies. We suspect that the dendritic morphology is the result of rapid nucleation and growth because of the lack of oleylamine as a surface-stabilizing agent. Interestingly, the Cu_7_PS_6_ phase could not be formed without oleylamine, suggesting that it also impacts precursor formation.

**Figure 7:**
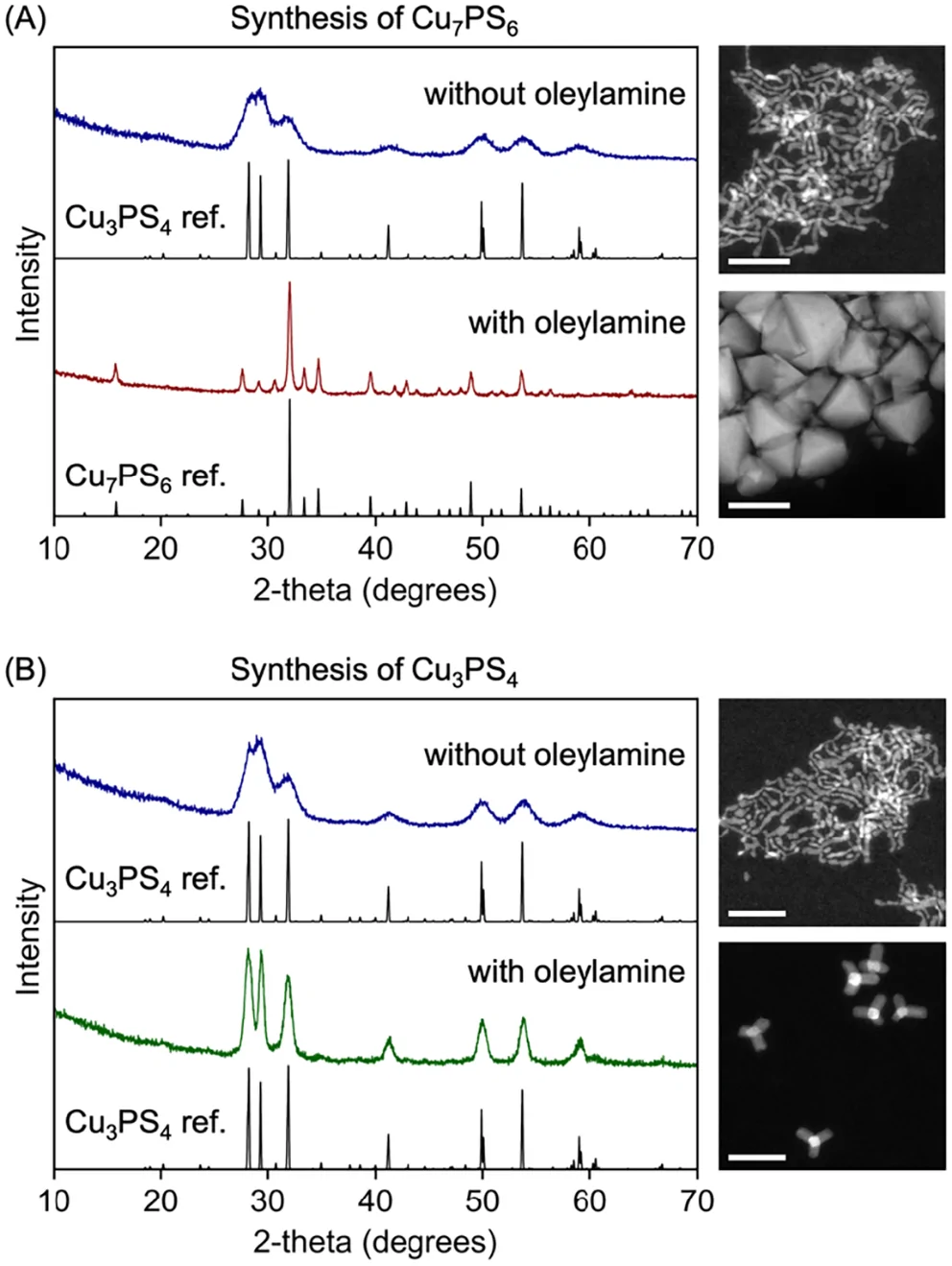
STEM images and XRD patterns highlighting the role of oleylamine in the synthesis of (A) Cu_7_PS_6_ and (B) Cu_3_PS_4_ nanocrystals. Scale bars are 50 nm.

Collectively, these experiments provide chemical insights into how colloidal copper phosphosulfide nanocrystals form, how phase selectivity (Cu_7_PS_6_ vs Cu_3_PS_4_) occurs, and how morphology evolves. All chemical components of the reaction are crucial to forming the desired products, with evidence of coupled interactions that influence reactivities and the availability of various species. The result is an ability to selectively synthesize Cu_7_PS_6_ as prism-shaped nanocrystals and Cu_3_PS_4_ as tetrahedral-shaped nanocrystals, which represent important additions to the library of synthetically accessible colloidal metal phosphosulfide nanocrystals.

## Conclusions

In this work, we demonstrated the synthesis of two distinct types of colloidal copper phosphosulfide nanocrystals: argyrodite Cu_7_PS_6_ and enargite (wurtzite-like) Cu_3_PS_4_. By considering the reaction chemistry, we found that careful control over the conditions, including reagent stoichiometry, concentration, and surface-stabilizing ligands, leads to the phase-selective formation of tetrahedron-shaped Cu_7_PS_6_ nanocrystals and tetrapod-shaped Cu_3_PS_4_ nanocrystals (Table S1). In the literature, these compositions are becoming increasingly useful as thermoelectrics, photovoltaic absorbers, and nonlinear optical materials,^3,7–17^ all of which could benefit in the future from accessibility as solution-processable nanocrystals. Additionally, the Cu^+^ cations in both bulk phases are known to exhibit moderate to high cationic conductivities, making them potentially useful in the form of nanocrystals as templates for cation exchange reactions that transform compositions while maintaining morphologies and crystal structure features.^34,35^ The ability to synthesize colloidal nanocrystals of Cu_7_PS_6_ is particularly interesting, as this compound is well-known in bulk to be a superionic conductor;^7,8,15,36^ such materials have been found to be useful as materials for solid-state electrolytes in batteries.^37^

## Supplementary Material



## Notes

Summary of synthesis reactions and additional HRTEM, HAADF-STEM, STEM-EDS, EDS, XRD, and UV–vis data (PDF)
