# Supplementary material for: Phase-Selective Synthesis of Copper Phosphosulfide Nanocrystals
Source: Inorg Chem. 2026 Oct 7;65(41):24483–91. doi: 10.1021/acs.inorgchem.6c04130 (PMC13685414; doi:10.1021/acs.inorgchem.6c04130)
Supplement: Supplementary file 1 [file ic6c04130_si_001.pdf]

SUPPORTING INFORMATION

Phase-Selective Synthesis of Copper Phosphosulfide Nanocrystals

Haley L. Young, Danushki N. Suriyawansa, and Raymond E. Schaak*

Department of Chemistry, Department of Chemical Engineering, and Materials Research Institute, Pennsylvania State University, University Park, PA 16802, United States

E-mail: res20@psu.edu

Table S1. Summary of copper phosphosulfide synthesis reactions

Copper phosphosulfide synthesis						
Cu:P ratio	CuCl (mmol)	P_2S_5 (mmol)	1-DDT	OLAM	Product	Data
1:1	0.125	0.063	9.5 mL	0.5 mL	Cu_7PS_6 prismatic	Figure 1
1:1	0.250	0.125			Cu_7PS_6 prismatic	
1:1	0.314	0.157			Cu_7PS_6 prismatic + Cu_3PS_4 tetrapods	
1:1	0.378	0.189			Cu_3PS_4 tetrapods	
1:1	0.500	0.250			Cu_3PS_4 tetrapods	
1:1	0.378	0.189	3.8 mL	0.2 mL	Cu_3PS_4 irregular and cubic	Figure 2A
3:1	0.750	0.125	9.5 mL	0.5 mL	Mixture of Cu_7PS_6 and $Cu_{2-x}S$	Figure 6
7:1	1.75				Mixture of Cu_7PS_6 and $Cu_{2-x}S$	
3:1	1.134	0.189	9.5 mL	0.5 mL	Mixture of Cu_3PS_4 and Cu_7PS_6	
7:1	2.646				Mixture of Cu_7PS_6 and $Cu_{2-x}S$	
1:1	0.250	0.125	9.5 mL	-	Cu_7PS_6, irregular and dendritic	Figure 7
1:1	0.378	0.189	9.5 mL	-		

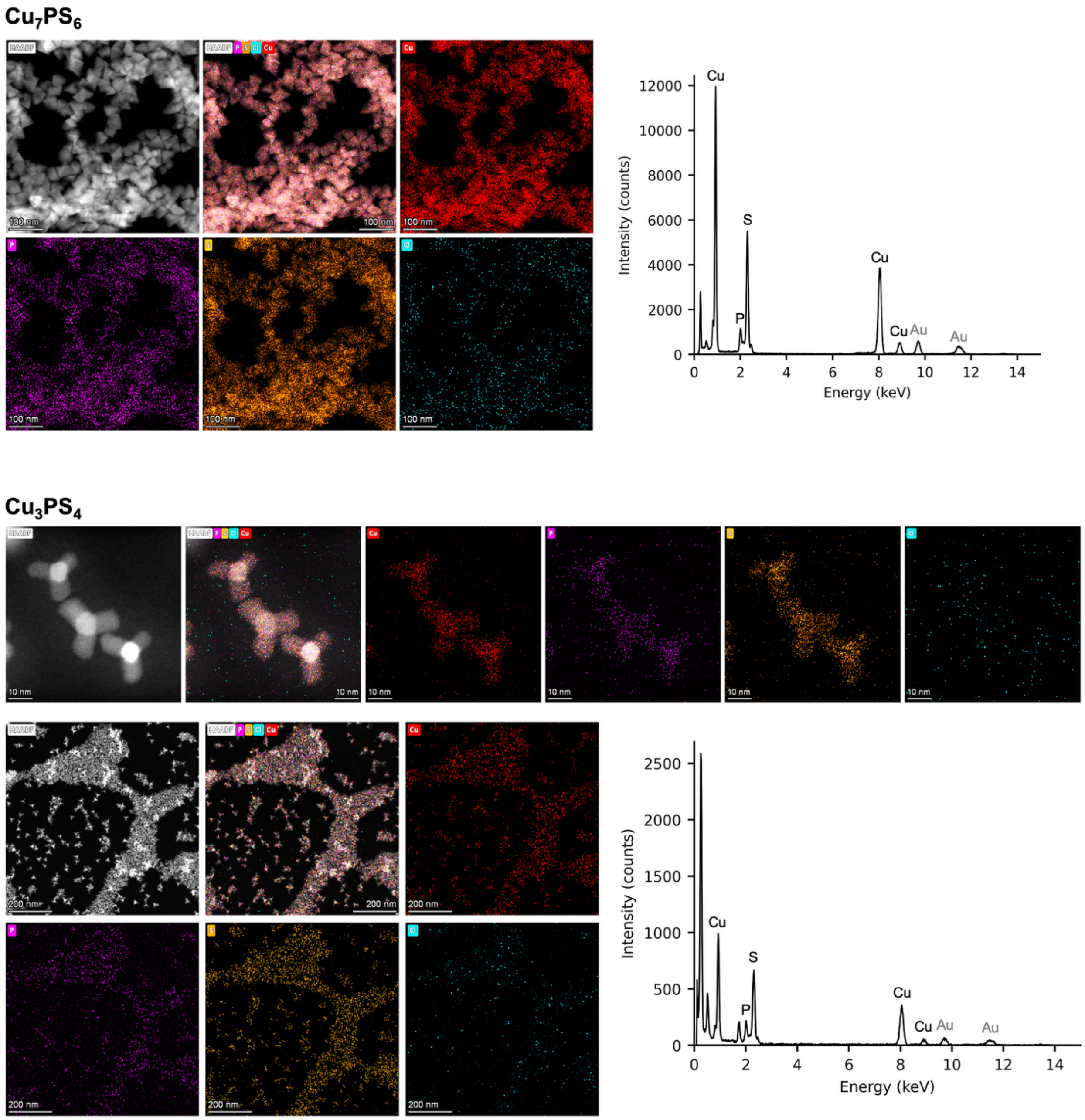

Figure S1. HAADF-STEM images and corresponding STEM-EDS elemental maps for Cu_7PS_6 and Cu_3PS_4 nanocrystals, along with ensemble EDS spectra. The elemental maps show Cu Kα (red), P Kα (purple), S Kα (orange), and Cl Kα (cyan). The Au is from the Au TEM grid and the unlabeled peaks correspond to adventitious C, N, O, and Si; no Cl was detected. For Cu_7PS_6, the Cu:S ratio is approximately 1:1, which is within typical experimental error of the 1.16:1 Cu:S ratio expected for Cu_7PS_6, while the P:S ratio is 1:5 (0.20), close to the expected ratio of 1:6 (0.17). For Cu_3PS_4, the Cu:S ratio is approximately 0.75, which is close to the expected ratio of 0.66, while the P:S ratio is 0.36, which is more phosphorus-rich than the value of 0.25 expected for Cu_3PS_4 and therefore may indicate the presence of a phosphorus-rich surface.

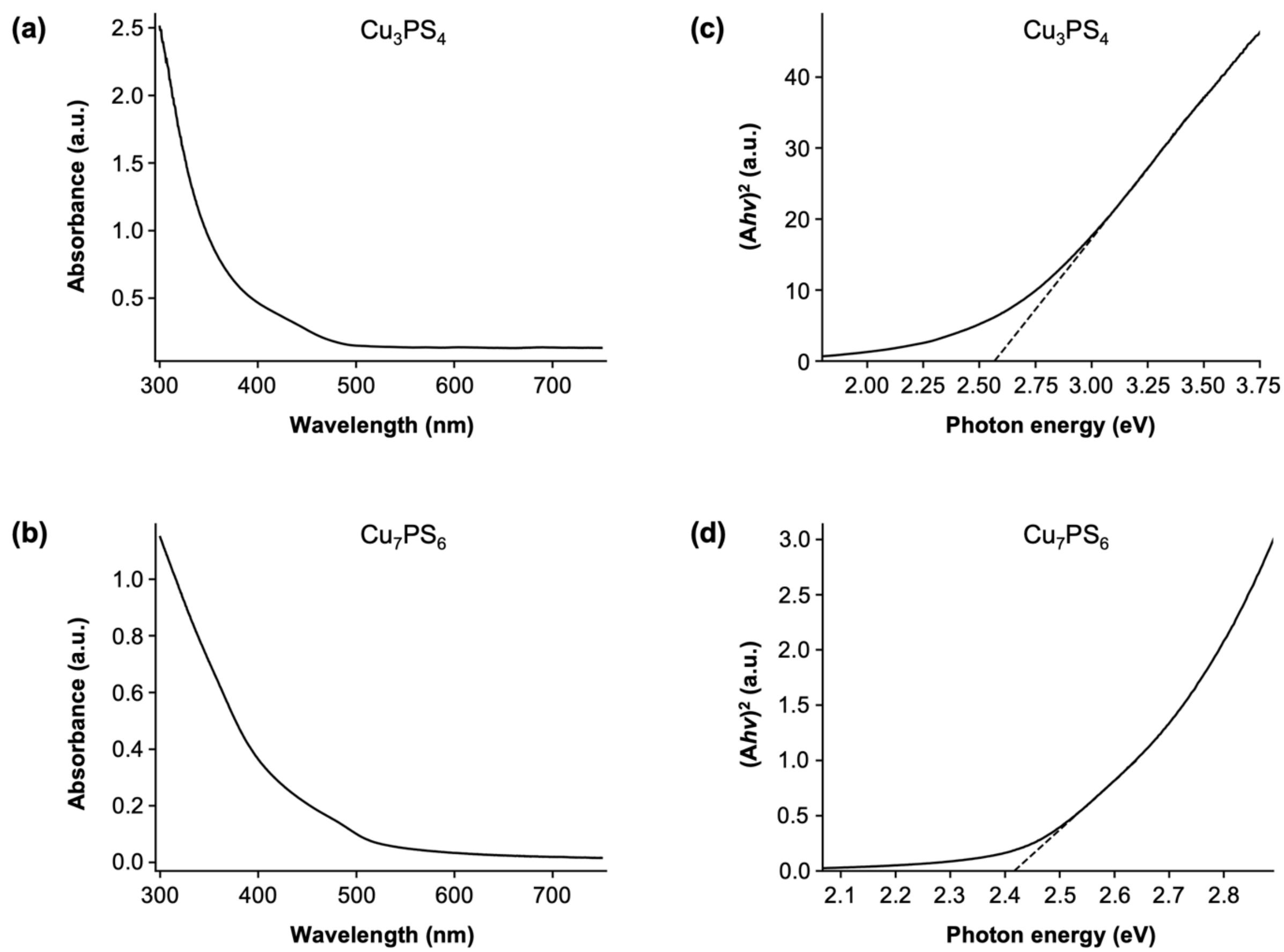

Figure S2. (a,b) UV-Vis absorption spectra and corresponding (c,d) Tauc plots for Cu_3PS_4 and Cu_7PS_6 nanocrystals.

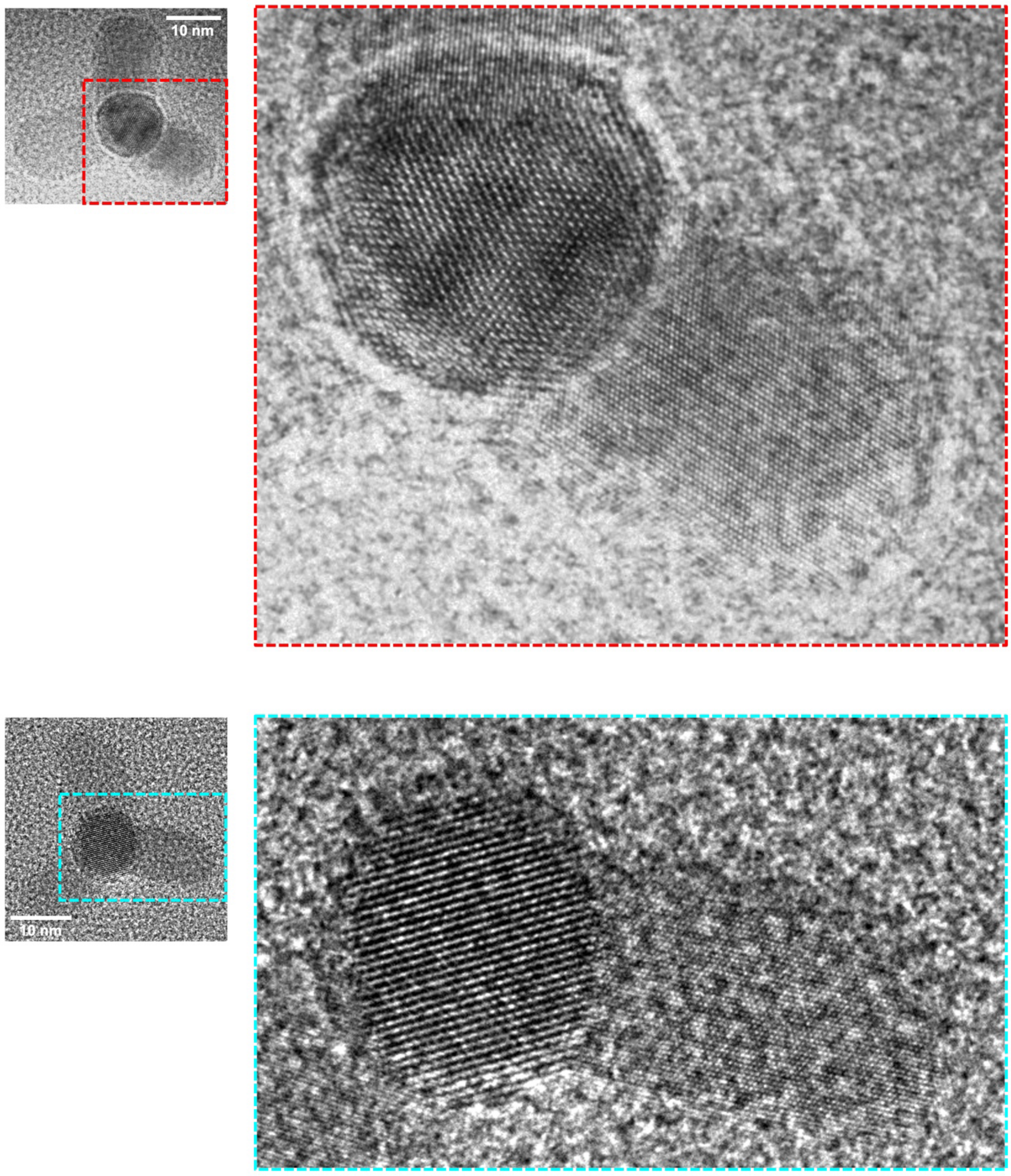

Figure S3. HRTEM images of additional Cu_3PS_4 tetrapods, showing the crystallinity of the arms that are oriented near-horizontal as well as the arm oriented vertically.

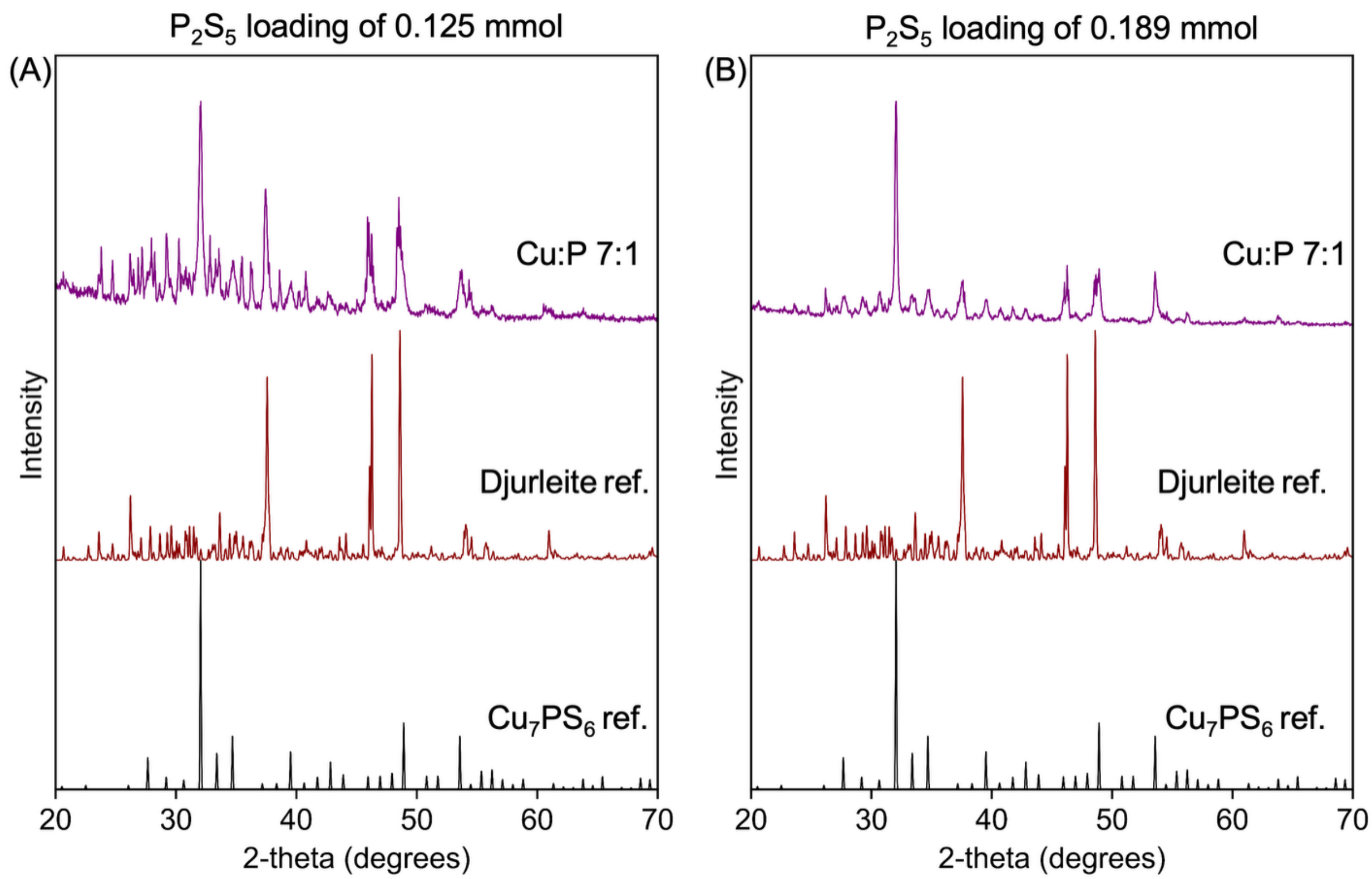

Figure S4. The presence of copper sulfide impurities was verified by XRD. As representative examples, the reaction product when a Cu:P ratio of 7:1 is used with a P_2S_5 loading of (A) 0.125 mmol and (B) 0.189 mmol was chosen. Here, the experimental pattern from Figure 6A and 6B are shown with the reference pattern of the predominate djurleite copper sulfide impurity. The reference pattern for djurleite was taken from Evans, H. T., The Crystal Structures of Low Chalcocite and Djurleite. *Zeitschrift fur Kristallographie*. **1979**, *150*, 299-320.
